# Association between intimate partner violence and utilization of facility delivery services in Nigeria: a propensity score matching analysis

**DOI:** 10.1186/s12889-019-7470-1

**Published:** 2019-08-17

**Authors:** Sanni Yaya, Nathali Gunawardena, Ghose Bishwajit

**Affiliations:** 1 The George Institute, University of Oxford, Oxford, UK; 2 Faculty of Sciences, University of Ottawa, Ottawa, Canada; 3 School of International Development and Global Studies, Faculty of Social Sciences, University of Ottawa, Ottawa, Canada

**Keywords:** Facility delivery, Intimate partner violence, Demographic and health survey, Global health, Nigeria

## Abstract

**Background:**

Intimate partner violence (IPV) has been shown to be associated with poor maternal healthcare utilisation and poor pregnancy outcomes. IPV can be seen both as the cause and result of low socioeconomic status and lack of maternal autonomy that can limit women’s access to resources and motivation necessary for seeking healthcare during pregnancy. This paper aims to study the relationship between intimate partner violence (IPV) and the utilisation of facility delivery services in Nigeria.

**Methods:**

We applied propensity score matching (PSM) approach to examine the relationship between intimate partner violence (IPV) and the utilisation of facility delivery services. PSM is a popular strategy for reducing sampling bias through balancing sample characteristics, a technique that mimics randomization on cross-sectional data. Data were collected from Nigeria DHS surveys conducted in 2008 and 2013. IPV was the main explanatory variable of interest for delivery at health facility which was defined as delivering at any health institution including health clinics.

**Results:**

PSM generated 20,446 cases distributed into two equal groups i.e. those who delivered at health facility versus those who did not. The prevalence of facility delivery in 2013 was 56.8% (95%CI 55.0–58.6) indicating a moderate increase from its 2008 level of 43.2% (41.4–45.0%). Lifetime prevalence of emotional, physical and sexual abuse was respectively 21.5%(95%CI 20.6, 22.4), 14.9% (14.2, 15.7) and 5.0% (4.6–5.4). In the multivariable analysis after adjusting for potential confounders, ever experiencing emotional abuse was associated increased odds of not delivering at a health facility. (AOR = 1.228, 95%CI, 1.095–1.679).

**Conclusion:**

Women experiencing emotional violence are less likely to use institutional delivery services, and hence are susceptible to increased risk of reproductive complications. IPV is a complex issue that needs to be tackled by introducing evidence based strategies contextually relevant to local sociocultural environment. Further studies are required to understand the roots of IPV and the pathways through which it hindrances healthcare utilisation among women.

## Background

Intimate partner violence (IPV) is among one of the most common types of violence against women [1–3]. It exists throughout the world [4] and is a major public health problem. Approximately 30% of women worldwide have reported physical or sexual abuse by a partner whom they have been in an intimate relationship with [4]. Prevalence of IPV in Sub-Saharan Africa is substantially high, with prevalence estimated between 20 and 70% [4]. Non-governmental organizations, World Health Organisation, and other organizations recognize the problem of IPV and have called on countries to take appropriate measures in reducing IPV against women [5]. Despite the calls made by these organizations, IPV remains rampant and continues to affect millions of women throughout the world [6]. Prevalence of violence against women in Nigeria varies with region and is estimated to be anywhere from 11 to 17% [7–11]. Because there are no standard methods to estimate IPV, there is a wide range in reported prevalence rates. Within Nigeria, violence against women is highly underreported [7–10].

IPV prevented the attainment of Millennial Development Goals including those pertaining to lowering maternal/child morbidity and mortality [5]. The Millennial Development Goals (MDG) 5 was targeted to improve maternal health, reduce maternal deaths, and create universal access to maternal or reproductive health services by 2015 [12]. The Sustainable Development Goals (SDGs) also place strong emphasis on promoting gender equality and women’s empowerment that can reduce the women’s vulnerability to IPV. Conversely, efforts to address IPV will also help achieving the gender violence related SDGs [1]. IPV can lead to complications in pregnancy including miscarriage, bleeding, anaemia and infection amongst others through direct and indirect mechanisms thus lowered the chances of meeting the Millennial Development Goals [5, 9, 13–16].

Although other studies have looked at the relationship between partner violence and maternal health [17], there have been few studies looking at the impact of IPV on utilisation of maternal health services in Nigeria. Furthermore, given the widespread prevalence of IPV, exploring the relationship between IPV and health facility delivery can be particularly useful for maternal health programs in the country. Therefore, this study used propensity score matching and data collected from Nigeria DHS surveys conducted in 2008 and 2013 to determine the relationship between intimate partner violence (IPV) and the utilisation of facility delivery services in Nigeria. For better understanding of the relationship between IPV and maternal health facility utilisation in Nigeria, the results can be used as a policy tool in order to design programs that will lower IPV and thus increase maternal health facility utilisation within the country.

## Methods

### The survey and sampling design

The Nigeria Demographic and Health Surveys (NDHS) of 2008 and 2013 were both implemented by the National Population Commission (NPC). Technical and financial assistance were given by Inner City Fund International which came through the USAID funded MEASURE DHS program. DHS collect information on a wide range of health topics including anthropometric, socioeconomic, demographic, family planning and domestic violence. The surveys are nationally representative and include men and women aged 15–49 years old and children under the age of 5 years residing in non-institutional settings. Participants in the surveys were sampled following a three-stage stratified cluster design using a list of enumeration areas (EAs) obtained from the Nigerian 2006 population census. EAs are units selected systematically from localities that constitutes the Local Government Areas (LGAs) – subdivisions of the 36 administrative states that are classified under six developmental zones in Nigeria. For the two NDHS used in this study, 38,948 and 33,385 women were respectively interviewed with a response rate of 98% for the 2013 NDHS and 97% for the 2008 NDHS. The sample selection strategy has been presented in Table 1 and details about the surveys have been published online on the main surveys’ reports.

**Table 1:** Sample selection strategy

Number of participants with information on place of delivery	Before matching	After matching
NDHS 2008	20,149	10,223
NDHS 2013	17,982	10,223
Total	38,131	20,446

### Variables

Outcome variable was location of most recent childbirth. This was measured by asking the respondent about the place of delivery for the most recent childbirth, and was dichotomised in the following way: (1) Institutional (For deliveries occurring at a Government hospital, District hospital, Private hospital/clinic, Private medical college hospital); and (2) Non-Institutional (For deliveries occurring at respondents’ or relatives’ homes, or in other nonprofessional facilities).

The explanatory variable of focus was three lifetime measures of IPV:

- Emotional: Ever any emotional violence/ Spouse ever humiliated her/ spouse ever threatened her with harm.
- Physical: Spouse ever punched with fist or something harmful spouse ever pushed, shook or threw something spouse ever slapped.
- Sexual: spouse ever forced other sexual acts when not wanted spouse ever physically forced sex when not wanted.

### Covariates

Year: 2008/ 2013; Age groups 15–19/ 20–24/ 25–29/ 30–34/ 35–39/ 40–44/ 45–49); type of place of residence: Urban/ Rural; Region: North Central/ North East/ North West/ South East/ South South/ South West; Religion: Christian/ Islam/ Other; Education: no education/ Primary/ Secondary/ Higher; Wealth index: poorest/ poorer/ middle/ richer/ richest; Husbands education: No education/ primary/ secondary/ higher; Sex of household: Male/ Female; Total children born: 1–2/ 3–4/ > 4; Has health insurance: No/ yes.

### Data analysis

Data were analysed with SPSS 24. Women who has a childbirth during past five years were included in the analysis. We used propensity score technique that simulates randomisation by matching the groups by outcome status (e.g. user vs non user of facility delivery service) for the predictor variables, which reduces the likelihood of bias in the treatment effect. The main advantage of this method is that it mimics certain characteristics of randomized controlled trials and thereby minimises the bias due to non-randomisation in observational studies. For this study, we used we used logistic regression as estimation algorithm and nearest neighbour matching as matching algorithm with tolerance level of 0.01%. At the first step of the analysis, basic sociodemographic characteristics of the participants were presented as percentages. Prevalence of IPV was calculated for the years 2008 and 2013. Following descriptive analysis, Chi-square (χ2) test was performed to check for the significant associations between the explanatory variables and place of delivery. Variables that were found to be significantly associated in the χ2 tests (at *p* < 0.25) were selected for final regression analysis. In the final step, binary logistic regression model was used to calculate the odds ratios (OR) of the associations between place of delivery and three types of IPV (physical, emotional, sexual).

## Results

In total 20,446 women were included in the study (Table 2). The prevalence of health facility delivery was 43.2% (95%CI = 41.4–45.0) in 2008 and rose to 56.8% (95%CI = 55.0–58.6) in 2013. Table 2 shows that the prevalence was higher among women in the age group of 25–29 years, in the urban areas, located in the South West region, had secondary level education, followers of Christianity, lived in the households with highest wealth quintile, had partners with secondary level education, were from male-headed households, had 1–2 children, had no health insurance.

**Table 2:** Sample description

Variables	*N* = 20,446			% of women delivered at health facility			*p*
	% of total, 95%CI			%, 95CI			<.0001
Year							
2008	46.6	44.9	48.3	43.2	41.4	45.0	
2013	53.4	51.7	55.1	56.8	55.0	58.6	
Age groups							<.0001
15–19	6.5	6.0	7.0	3.6	3.2	4.1	
20–24	20.5	19.8	21.2	16.9	16.0	17.8	
25–29	28.5	27.8	29.2	29.3	28.3	30.3	
30–34	21.1	20.5	21.8	24.2	23.3	25.1	
35–39	14.1	13.6	14.6	16.2	15.4	17.0	
40–44	6.8	6.4	7.2	7.4	6.8	8.0	
45–49	2.5	2.3	2.8	2.4	2.1	2.7	
Type of place of residence							<.0001
Urban	35.9	34.3	37.6	54.4	52.6	56.2	
Rural	64.1	62.4	65.7	45.6	43.8	47.4	
Region							<.0001
North Central	19.3	18.4	20.3	22.9	21.5	24.3	
North East	20.9	19.5	22.3	10.2	9.0	11.5	
North West	25.7	24.3	27.2	8.4	7.4	9.5	
South East	9.3	8.6	10.0	16.4	15.3	17.7	
South South	9.6	8.9	10.3	13.9	12.8	15.0	
South West	15.2	14.3	16.2	28.3	26.7	29.9	
Religion							<.0001
Christian	44.6	42.8	46.5	64.8	62.9	66.7	
Islam	53.8	51.9	55.6	34.0	32.1	35.9	
Other	1.6	1.4	1.9	1.2	0.9	1.4	
Education							<.0001
no education	39.2	37.6	40.8	15.0	13.9	16.2	
Primary	23.1	22.2	24.1	23.8	22.7	25.0	
Secondary	29.7	28.6	30.9	46.1	44.7	47.5	
Higher	7.9	7.3	8.5	15.1	14.1	16.3	
Wealth index							<.0001
Poorest	20.3	18.9	21.8	5.3	4.6	6.1	
Poorer	20.5	19.4	21.5	11.2	10.2	12.3	
Middle	20.1	19.1	21.2	19.2	18.0	20.4	
Richer	19.8	18.7	20.9	28.8	27.3	30.3	
Richest	19.3	18.1	20.5	35.5	33.6	37.5	
Husbands education							<.0001
No education	25.2	23.7	26.8	10.9	10.0	11.9	
Primary	24.0	23.1	25.0	21.1	20.1	22.1	
Secondary	34.6	33.5	35.7	44.5	43.2	45.8	
Higher	16.2	15.3	17.0	23.5	22.3	24.8	
Sex of household							
Male	90.0	89.4	90.6	86.4	85.5	87.3	
Female	10.0	9.4	10.6	13.6	12.7	14.5	
Total children bor							<.0001
1–2	36.8	36.0	37.6	41.4	40.3	42.5	
3–4	30.9	30.2	31.7	32.1	31.1	33.1	
> 4	32.3	31.5	33.1	26.5	25.5	27.6	
Has health insurance							<.0001
No	97.8	97.4	98.1	96.1	95.5	96.7	
Yes	2.2	1.9	2.6	3.9	3.3	4.5	
Emotional IPV							
No	78.5	77.6	79.4	77.3	76.1	78.4	<.0001
Yes	21.5	20.6	22.4	22.7	21.6	23.9	
Physical IPV							<.0001
No	83.9	83.1	84.7	81.4	80.4	82.4	
Yes	16.1	15.3	16.9	18.6	17.6	19.6	
Sexual IPV							<.0001
No	95.0	94.6	95.4	95.2	94.6	95.7	
Yes	5.0	4.6	5.4	4.8	4.3	5.4	

N.B. *p*-values generated from Chi-square tests

**Fig. 1:**
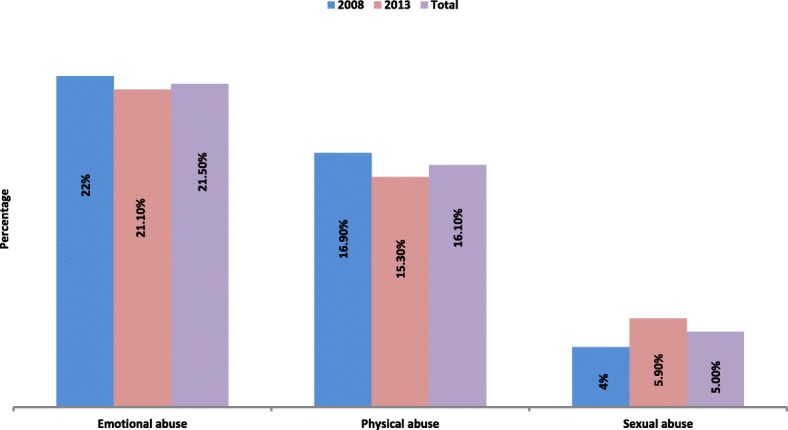
Prevalence of abuse among Nigerian women

Figure 1 shows the prevalence of three types of IPVs. Overall, 21.5, 16.1 and 5% of the women reported ever experiencing emotional, physical and sexual violence.

**Fig. 2:**
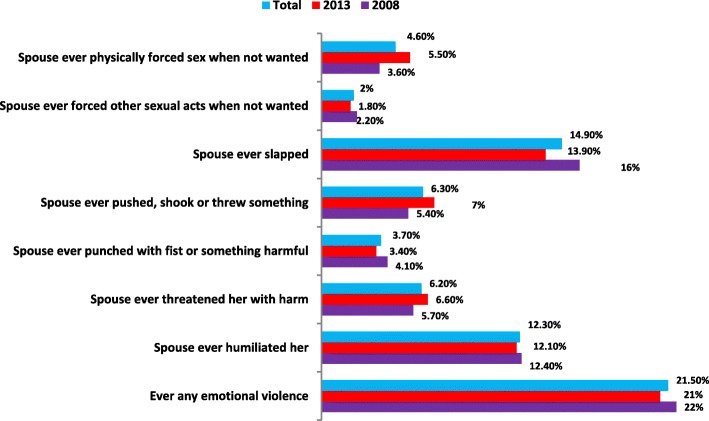
Prevalence of IPV among Nigerian women aged 15–49 years

In Fig. 2, about 21.5% reported lifetime experience of emotional violence. The overall proportions of women who had ever been slapped was approximately 14.9%, those who had ever been humiliated was 12.3%. However, lifetime experience of punch or fist with something harmful, pushed shook or threw something, ever forced other sexual acts when not wanted and physically forced sex when not wanted were relatively low among Nigerian women.

Results of regression analysis on the association between place of delivery and three types of IPV were presented in Table 3. In the adjusted models, experiencing emotional (OR = 1. 228, 95%CI = 1.095–1.679) and physical violence (OR = 1.477, 95%CI = 1.128–2.072) were found to be significantly associated with higher odds of not delivering at health facility. That for sexual violence also showed higher odds of not delivering at health facility, however the association was not statistically significant (OR = 1.123, 95%CI = 0.901–1.398).

**Table 3:** Odds ratios of the association between types of IPV and not delivering at health facility in Nigeria

Type of IPV	^a^COR, 95%CI			AOR, 95%CI		
Reference category = No						
Ever any emotional violence	0.970	0.827	1.136	0.995	0.847	1.168
Spouse ever humiliated her	1.031	0.880	1.208	0.981	0.814	1.183
Spouse ever threatened her with harm	1.944	1.635	2.311	**1.515**	**1.217**	**1.887**
Emotional abuse combined	1.029	0.925	1.145	**1.228**	**1.095**	**1.679**
Spouse ever punched with fist or something harmful	1.150	0.933	1.417	0.993	0.761	1.296
Spouse ever pushed, shook or threw something	1.447	1.223	1.711	0.839	0.670	1.051
Spouse ever slapped	1.187	1.047	1.346	1.087	0.929	1.272
Physical abuse combined	1.509	1.352	1.684	1.044	0.913	1.195
Spouse ever forced other sexual acts when not wanted	1.430	1.086	1.882	**1.477**	**1.128**	**2.072**
Spouse ever physically forced sex when not wanted	1.333 1.090		1.629	1.106	0.860	1.422
Sexual abuse combined	1.380	1.164 1.636		1.123	0.901	1.398

N.B. ^a^ = unadjusted, ^a^ = adjusted for Year, Age groups, Type of place of residence, Region, Religion, Education, Wealth index, Husbands education, Sex of household, Total children born, Has health insurance. *COR* Crude odds ratio, *AOR* Adjusted odds ratio. Significant AORs are shown in Bold

## Discussion

Through this study we were able to determine that women experiencing emotional violence in Nigeria may under-utilize institutional delivery services, and hence are susceptible to increased risk of reproductive complications.

PSM generated 20,446 cases distributed into two equal groups i.e. those who delivered at health facility versus those who did not. The prevalence of facility delivery in 2013 was slightly below half indicating a moderate increase from its 2008 level. A major finding of the study was that most women who delivered at a health care facility were 25–29 years of age, lived in an urban area, were from the South West region, were Christian, had secondary level education, were from the richest or second richest wealth status, and had no health insurance. Other studies have found that the age of women, their family setting, and education level was associated with the level of IPV they experienced [9, 13, 18, 19]. Lifetime prevalence of emotional abuse was highest, physical and sexual abuses were least. Our findings for the prevalence of emotional violence is similar to that of another study conducted in Nigeria that found that 22.4% of women had experienced at least one type of emotional violence by a male partner [20].

In the multivariable analysis after adjusting for potential confounders, ever experiencing emotional abuse was associated increased odds of not delivering at a health facility. Our findings are consistent with another study that found that women who experience emotional violence are less likely to utilise skilled antenatal care, facility delivery and skilled assistance [21]. Another study conducted in Nigeria also found that women who underwent emotional IPV were less likely to use antenatal care [22]. These findings suggest that emotional violence may be playing a less noticeable yet important role in poor utilisation of maternal health services. The possible mechanism by which IPV can reduce the access to healthcare services among women is through affecting their psychosocial situation and health promoting behaviour, which are necessary preconditions for uptake of maternal health care services. In order to boost maternal health services utilisation more attention is needed to be paid to emotional violence. Most studies on IPV have focussed on physical and sexual IPV and their outcomes [23], while there is still little known on the relationship between emotional IPV and utilization of maternal health care services.

In an effort to improve reproductive health in Nigeria, IPV and women’s health right issues are being highlighted in policy making in the country [24]. In many states within the country, The Gender Policy for the Nigeria Police Force (2010) and Gender-based Violence (Prohibition) Law are administered in order to reduce violence against women and as a result, improve women’s health [25]. Despite these initiatives, IPV is still persistent globally [6]. Having a better understanding of the impact of IPV on the choice of maternal health care facility is important. A potential solution can be to lower IPV is by making it, as well as its negative consequences, aware to the public through public enlightenment [26, 27]. Attitude change is also paramount to reducing IPV. Attitude changes can be made by empowering women, promoting gender equality, education, and advocacy as described by the Millennial Development Goals [28–30].

## Strengths and limitations

The evidence gathered from this study filled an important gap in research and increased our understanding of the association between IPV and utilization of maternal health care facilities. This study also had reduced bias by using applied propensity score matching (PSM) which is used as a popular strategy for reducing sampling bias by balancing sample characteristics, a technique that mimics randomization on cross-sectional data. However, PSM is not without its limitation. An inherent issue is that the investigators do not have control over the exposure to participants and oftentimes it is unlikely that the outcome will be experienced by them for the given context. One important limitation of the study is the possible underreporting of IPV by the study population. Violence is often underreported and there is a chance that some women in the study failed to report IPV that they have experienced [31, 32]. We also used lifetime prevalence of IPV which might have influence the association as the data were collected for the most recent childbirth. Stigmatization as well as personal (embarrassment, economic dependence) and societal reasons (imbalanced power relations between men and women in society) can cause women to underreport IPV [33–35].

## Conclusion

Intimate partner violence exists in Nigeria. All types of intimate partner violence but especially emotional violence may cause Nigerian women to under-utilise institutional delivery services, and hence making them susceptible to increased risk of reproductive complications. Healthcare policies and programs connected to maternal empowerment, improved behaviour change communication could help address persistent IPV in Nigeria. Further studies are required to understand the causes of IPV and possible pathways by which hinders healthcare utilisation among women as to proffer long-term solutions.

## Data Availability

Data for this study were sourced from Demographic and Health surveys (DHS) and available here: http://dhsprogram.com/data/available-datasets.cfm.

## Abbreviations

DHS: Demographic and Health Survey
EAs: Enumeration Areas
IPV: Intimate Partner Violence
LGAs: Local Government Areas
PSM: Propensity Score Matching
